# Long-term outcome after management of pilocytic astrocytoma in the posterior fossa in a pediatric population

**DOI:** 10.1016/j.ibneur.2022.10.001

**Published:** 2022-10-07

**Authors:** Alejandro N. Santos, Celina Dieckmann, Laurèl Rauschenbach, Marvin Darkwah Oppong, Thiemo Florin Dinger, Cornelius Deuschl, Stephan Tippelt, Gudrun Fleischhack, Börge Schmidt, Daniela Pierscianek, Ramazan Jabbarli, Karsten H. Wrede, Oliver Müller, Ulrich Sure, Philipp Dammann

**Affiliations:** a Department of Neurosurgery and Spine Surgery, University Hospital Essen, Essen, Germany; b Institute of Diagnostic and Interventional Radiology and Neuroradiology, University Hospital Essen, Germany; c Pediatrics III, University Hospital of Essen, Essen, Germany; d Institute for Medical Informatics, Biometry and Epidemiology, University Hospital of Essen, Essen, Germany; e Department of Neurosurgery and Spine Surgery, University Hospital Essen, Essen, Germany; Current address: Department of Neurosurgery, Hospital Dortmund, Dortmund, Germany

**Keywords:** CN, cranial nerve, FU, follow-up, PA, pilocytic astrocytoma, mRS, modified Rankin scale, Pediatric, Pilocytic astrocytoma’s, Surgical management, Outcomes, Risk factors

## Abstract

**Background and purpose:**

To assess the impact of posterior fossa pilocytic astrocytoma (PA) removal in pediatric patients, with special focus on postoperative neurological outcome after repeated surgery for tumor remnants.

**Methods:**

Our institutional database was screened for patients with PA treated between 2000 and 2019. Patients ≤ 18 years of age with complete clinical records, preoperative contrast enhanced magnetic resonance imaging (MRI) and postoperative follow-up time of ≥ 6 months were suitable for study inclusion. Functional outcome was quantified with the modified Ranking Scale (mRS) score and assessed at admission, at discharge and at every follow-up investigation. Predictors of hydrocephalus, cranial nerve deficits and tumor recurrence were evaluated.

**Results:**

A total of 57 pediatric patients with a mean age of 7.7 ± 4.8 years were included in the analysis. 27 (47.3%) children suffered from hydrocephalus at diagnosis, out of which 19 (33.3%) required a subsequent VP-Shunt. 22 (39.3%) patients had a partial resection, of which 9 (40.9%) went through second-look surgery. 2 patients with initially radiological confirmation of complete resection, had a tumor recurrence at FU and needed second-look surgery. Among the children requiring second-look surgery, 7 (63.6%) had a complete resection. Favorable outcome (mRS≤2) after initial and second-look surgery was observed in 52 patients (91.2%). Univariate analysis identified tumor location in the floor of the 4th ventricle (p = 0.030), and repeated surgery for tumor remnant removal (p = 0.043) as predictors for post-operative cranial nerve deficits. Multivariate analysis confirmed this independent association. The incidence of tumor recurrence occurred more often in patients with previous partial resection (p = 0.009) as well as in lesions located in the cerebellar peduncles (p = 0.043). Partial resection remained an independent predictor after multivariate logistic regression analysis (p = 0.045).

**Conclusions:**

Incomplete resection of posterior fossa PA is a risk factor for tumor recurrence and repeated surgery to remove tumor remnants increases the risk of new postoperative deficits. Thus, the risk of iatrogenic deterioration due to second look surgery should be implemented in the primary pre- and intraoperative decision-making.

## 1 Introduction

Pilocytic astrocytoma (PA) in the posterior fossa is one of the most common tumor entities in pediatric patients although the overall incidence in the whole population remains low (Villanueva et al., 2019, Donofrio et al., 2020, Rickert and Paulus, 2001). The current standard of care for such lesions is surgery with maximal extent of resection, sometimes followed by radiotherapy in case of incomplete resection and recurrent tumor growth (Villanueva et al., 2019, Park et al., 2019, Palma et al., 2004). Being a benign glial tumor (WHO grade I), the 5-year survival rate for PA in general is estimated to be ≈ 97% (Segal and Karajannis, 2016), which is highly influenced by the extent of surgical resection (Park et al., 2019, Fernandez et al., 2003). Symptoms leading to diagnosis such as ataxia, sensorimotor or cranial nerve (CN) deficits appear mainly due to space occupying tumor growth, while drowsiness, nausea and dizziness are oftentimes caused by obstruction of the ventricular system leading to hydrocephalus (Salles et al., 2020, Won et al., 2020). Although being a well-established procedure, surgical treatment of posterior fossa PA can be technically challenging and associated with severe postoperative morbidity due to the complex anatomy, making gross total resection challenging (Palma et al., 2004, Pollack, 1999 May, Ogiwara et al., 2012). Given the low incidence of tumor occurrence, studies surrounding surgical complications and long-term follow-up are rare (Palma et al., 2004, Won et al., 2020, Collins and Pollack, 2020, Benesch et al., 2006). Furthermore, there is a lack of trials investigating the benefit of repeated surgery to remove tumor remnants after incomplete resection. Notably, the existing studies are heterogeneous and usually comprise only a small number of patients (Palma et al., 2004, Benesch et al., 2006, Ait Khelifa-Gallois et al., 2015, Kristiansen et al., 2019).

The purpose of this study was to give a detailed descriptive mono-center experience with a special focus on long-term postoperative outcome after second-look surgery.

## 2 Methods

### 2.1 Data collection

This study was conducted at our tertiary referral hospital, in accordance with the principles expressed in the Declaration of Helsinki and the guidelines of an approving institutional review board (14–5751-BO and 19–8662-BO). The local ethics committee approved the use of the anonymized data for further analysis. A retrospective cross-sectional study was performed of all consecutive patients admitted to our department between 2000 and 2019 that fulfilled the following inclusion criteria: aged between 0 and 18 years; new-onset and histologically confirmed PA with infratentorial tumor localization; went through surgical removal of the lesion; postoperative follow-up of ≥ 6 months after surgical removal. Clinical baseline data of patients (sex, age at surgery, need of an external ventricular drainage (EVD) or ventriculoperitoneal shunt (VP-Shunt), second removal after partial resection and/or tumor progression/recurrence, postoperative radiotherapy and/or chemotherapy), as well as histological and molecular results focused on B-RAF gene and its possible molecular alteration (B-RAF V600E gene mutation, B-RAF–KIAA1549 gene translocation, etc) were obtained based on medical charts. Subsequent surgical resection or second-look surgery regarded all patients going through a new surgery to extract either a tumor remanent (seen at early post-operative MRI), or after showing progression/recurrence at follow-up MRI (generally at 3 months follow-up or sooner according to symptoms). Imaging data was assessed using pre- and post-operative MRI scans (tumor size, tumor composition (solid, cystic or mixed), hydrocephalus at diagnosis (based on Evans’ index), tumor location within the posterior fossa, extent of resection). Location of the tumor was selected among 8 anatomical regions being exophytic, vermis, cerebellar hemisphere, cerebellar peduncle, roof of 4th ventricle, floor of 4th ventricle, obex, foramen luschka. Tumors were often located in more than one anatomical region. Size of the lesion (in mm^3^) was obtained using Horos DICOM medical image viewer (3^d^ Version, Horos Project). Tumor mass was identified using triplanar reconstructed T1-contrast enhanced MP-RAGE /T2-weighted sequences. Functional outcome was assessed using the modified Rankin Scale (mRS). Follow-up was performed in a multidisciplinary standardized setting gathering a specialist Neurosurgeon and a specialist Pediatrician. Early post-operative MRI was defined as MRI within the first 72 h post-operatively. A minimum one-point increase on the mRS score compared to the preoperative score at the time of the interview was defined as neurological deterioration. Favorable outcome was defined as an mRS score of ≤ 2.

### 2.2 Statistical analysis

We used SPSS 26 (IBM, Armonk, NY, USA) for all statistical analyses. Nominal data were expressed as absolute numbers and valid percent and continuous variables were expressed as mean and standard deviations. Univariate analyses were performed to determine predictors regarding post-operative clinical outcome. For dichotomized variables, the Chi-Square test (sample size >5) or the Fisher exact test (sample size ≤5) were used. Continuous variables were tested with the Student’s t-Test (normally distributed data) or Mann-Whitney-U test (non-normally distributed data). Risk factors associated with hydrocephalus at diagnosis, post-operative cranial nerve (CN) deficit at last FU, as well as tumor progression were assessed by calculating odds ratios (ORs) and 95% confidence intervals (95% CIs) using logistic regression models including each potential predictor separately, as well as a logistic regression model including all significant risk factors. Results were considered statistically significant at an alpha-level of 0.05.

## 3 Results

### 3.1 Children demographics and outcomes

A total of 57 pediatric patients were included in the analysis. Mean age was 7.77 ± 4.86 years, and 24 individuals (42.1%) were female. Lesion’s histology was confirmed by local neuropathologists working in our tertiary referral center. The PA were rather heterogeneously distributed within the posterior fossa, with usually tumors located in more than one anatomical region. The most common involved location was the vermis accounting for 32 patients (56.1%) followed by the roof of the 4th ventricle accounting for 27 patients (47.3%). 14 (24.5%) PA were cystic, 18 (31.5%) solid and 17 (29.8%) mixed. The mean tumor size was 38.74 ± 26.64 mm^3^. 27 (47.3%) children suffered from hydrocephalus at diagnosis, out of which 19 (33.3%) required a subsequent VP-Shunt. 22 (39.3%) patients had a partial resection, of which 9 (40.9%) went through second-look surgery. 4 (18.2%) of them went through direct successive new removal of the remnant, and 5 (22.7%) after tumor progression seen at FU. Out of these 13 patients with a postoperative remnant that did not go through second-look surgery, only 3 (23.1%) showed a progression at last FU. Additionally, 2 patients with initially radiological confirmation of complete resection, had a tumor recurrence at FU and needed second-look surgery. Among the 11 children requiring second-look surgery, 7 (63.6%) finally had a complete resection. Reasons leading to second-look surgery were tumor progression/recurrence seen in 5 patients, as well as partial resection seen at early postoperative MRI within 72 h for 4 patients. Median FU time after surgery was 74 ± 57.5 months. At admission, 46 patients (80.7%) were in good clinical condition (mRS≤2). Favorable outcome (mRS≤2) after PA resection was observed in 52 patients (91.2%) and most patients (85.9%) revealed improved or unchanged scores. Moreover, molecular analysis was performed in 19 (33.3%) of patients. B-RAF–KIAA1549 gene translocation was found in 15 individuals (78.9%). Only 1 pediatric patient had a BRAF V600E gene mutation. This patient went through postoperative combined chemoradiotherapy due to tumor recurrence. Univariate analysis revealed no statistically significant influence of clinical baseline data on functional outcome at last FU. Namely, second-look surgery (OR = 0.87, 95% CI = 0.77–0.97, p = 0.579), preoperative hydrocephalus (OR = 3.48, 95% CI = 0.36–33.73, p = 0.369), postoperative radiotherapy (OR = 0.86, 95% CI = 0.80–0–98, p = 1.00) or postoperative chemotherapy (OR = 0.87, 95% CI = 0.79–9.74, p = 1.00) had no influence on postoperative functional outcome. Detailed cohort characteristics are summarized in Table 1.

**Table 1:** Univariate analysis of predictors for functional postoperative outcome.

Parameter	mRS same or improved (n = 51, 89.4%)	mRS worse (n = 6, 10.5%)	p-value	OR	95%CI
Age (years), mean ± SD	7.6 ± 4.7	7 ± 3.9	0.789^a^	n/a	n/a
Female sex, n (%)	21 (41.2%)	2(33.3%)	1.000^b^	0.67	0.11–3.99
Median FU, n ± SD	63.5 ± 53.5	93 ± 43.4	0.686^a^	n/a	n/a
Lesion location, n (%)* 1. Exophytic 2. Vermis 3. Cerebellar Hemisphere 4. Cerebellar Peduncle 5. Roof of 4th Ventricle 6. Floor of 4th Ventricle 7. Obex 8. Foramen Luschka	4 (9.3%) 28 (65.1%) 17 (39.5%) 11 (25.6%) 23 (53.5%) 9 (20.9%) 10 (23.3%) 9 (20.9%)	1 (20%) 4 (80%) 1 (20%) 3 (60%) 4 (80%) 3 (60%) 1 (20%) 0 (0%)	0.438^b^ 0.652^b^ 0.637^b^ 0.140^b^ 0.369^b^ 0.092^b^ 1.000^b^ 0.568^b^	2.44 2.14 0.38 4.36 3.48 5.67 0.83 0.79	0.28–27.43 0.22–20.94 0.04–3.72 0.64–29.64 0.36–33.73 0.82–32.20 0.08–8.25 0.68–0.92
Lesion composition, n (%)” 1. Cystic 2. Solid 3. Mixed	12 (27.4%) 18 (40.9%) 14 (31.8%)	2 (40%) 0 (0%) 3 (60%)	0.204^b^	n/a	n/a
Tumor size (mm^3^), mean ± SD	38.9 ± 26.3	45.1 ± 31.7	0.662^a^		
Hydrocephalus at diagnosis, n(%)*	23 (53.5%)	4 (80%)	0.369^b^	3.48	0.36–33.73
EVD, n (%)* *	27 (55.1%)	4 (66.7%)	0.686^b^	1.63	0.27–9.74
VP-Shunt, n (%)* *	16 (32%)	3 (50%)	0.405^b^	2.06	0.37–11.38
Partial resection, n (%)* *	17 (34.7%)	4 (66.7%)	0.188^b^	3.76	0.63–22.69
Second-look surgery, n(%)* *	10 (20.4%)	1 (16.7%)	0.579^b^	0.87	0.77–0.97
Radiotherapy, n (%)^	2 (4.1%)	0 (0%)	1.000^b^	0.86	0.80–0–98
Chemotherapy, n (%)^	5 (10.4%)	0 (0%)	1.000^b^	0.87	0.79–9.74
mRS preoperative, n (%) 1. 0 2. 1 3. 2 4. 3 5. 4 6. 5 7. 6	9 (18%) 23 (46%) 8 (16%) 6 (12%) 4 (8%) (0%) (0%)	3 (50%) 2 (33.3%) 1 (16.7%) 0(0%) 0(0%) 0(0%) 0(0%)	0.408^a^	n/a	n/a
mRS last FU, n (%) 1. 0 2. 1 3. 2 4. 3 5. 4 6. 5 7. 6	29 (58%) 15 (30%) 4 (8%) 2 (4%) 0 (0%) 0 (0%) 0 (0%)	0(0%) 2(33.3%) 3(50%) 0(0%) 1(16.7%) 0(0%) 0(0%)	**< 0.002**^a^	n/a	n/a

Univariate analysis of demographic, clinical, and anatomic factors for association with functional postoperative outcome (changes in mRS and Karnosky-index between admission and final follow-up). All patients with a last follow-up of ≥ 6 months included. a Student’s t-test or Mann-Whitney-U test. b Chi-Square test or Fisher exact test.

VP-Shunt: ventriculoperitoneal shunt. EVD: external ventricular drainage. mRS: modified Rankin Scale. n/a: not applicable.

* 9 patients missing; ”8 patients missing; * *2 patients missing; ^3 patients missing; ^^ 1 patient missing

### 3.2 Predictors of post-operative cranial nerve deficits

Univariate logistic regression analysis identified tumor localization in the floor of the 4th ventricle (OR = 5.54, 95% CI = 1.18–26.07, p = 0.030), as well as second-look surgery (OR = 4.41, 95% CI = 1.05–18.51, p = 0.043) as predictors for presenting post-operative cranial nerve deficits at last FU. Both parameters remained independently significant after multivariate logistic regression analysis (adjusted [a]OR = 16.83, 95% CI = 1.71–166.58, p = 0.016; aOR = 17.93, 95% CI = 1.66–193.53; p = 0.017). (Table 2, Table 3).

**Table 2:** Univariate Logistic Regression Analysis: Baseline characteristics and risk of Cranial Nerve deficit at last FU.

Parameter	p-value	OR	95%CI
Lesion location in Floor of 4th Ventricle	**0.030**	5.54	1.18–26.07
Second-look surgery	**0.026**	5.28	1.22–22.87

OR: odds ratio. CI: Confidence Interval

**Table 3:** Multivariate Logistic Regression Analysis: Baseline characteristics and risk of Cranial Nerve deficit at last FU.

Parameter	p-value	aOR	95%CI
Lesion location in Floor of 4th Ventricle	**0.015**	17.09	1.72–169.85
Second-look surgery	**0.012**	22.73	2.01–256.39

aOR: adjusted odds ratio. CI: Confidence Interval

### 3.3 Predictors of tumor progression and/or recurrence

Univariate logistic regression analysis identified tumor localization in the cerebellar peduncles (OR = 4.35, 95% CI = 1.05–18.03, p = 0.043), as well as partial resection (OR = 6.92, 95% CI = 1.61–29.80, p = 0.009) as predictors for tumor progression. Incomplete tumor resection remained an independent predictor after multivariate logistic regression analysis (aOR = 5.46, 95% CI = 1.12–26.52, p = 0.035). (Table 4, Table 5).

**Table 4:** Univariate Logistic Regression Analysis: Baseline characteristics and risk of Tumor progress.

Parameter	p-value	OR	95%CI
Lesion located in Cerebellar Peduncles	**0.043**	4.35	1.05–18.03
Partial resection	**0.009**	6.92	1.61–29.80

OR: odds ratio. CI: Confidence Interval

**Table 5:** Multivariate Logistic Regression Analysis: Baseline characteristics and risk of Tumor progress.

Parameter	p-value	OR	95%CI
Lesion located in Cerebellar Peduncles	0.224	2.62	0.56–12.40
Partial resection	**0.035**	5.46	1.12–26.52

OR: odds ratio. CI: Confidence Interval

### 3.4 Tumor remnants as identified on early post-operative MRI

Suspicion of a partial resection seen on early post-operative MRI was significantly associated with tumor remnant at last FU (OR = 0.11, 95% CI = 0.04–0.28, p < 0.001). Binary classification testing was performed to assess the predictive value of partial resection seen on early post-operative MRI. The sensitivity and specificity were 94.4% and 89.7%, respectively. (Table 6). Although specificity was not 100%, no statistically significant difference was seen between both groups (OR = 1.01, 95% CI = 0.14–8.02, p = 1.00).

**Table 6:** Predictive value of partial resection seen at early postoperative MRI after surgery.

	Gross total resection at early MRI	Partial resection at early MRI	Total	OR [95%CI]	p
No tumor remnant at last FU	N = 35 (89.7%) (TN)	N = 1 (5.6%) (FP)	N = 36 (63.2%)	16.54 [2.40–108.84]	**< 0.001**
Tumor remnant at last FU	N = 4 (10.3%) (FN)	N = 17 (94.4%) (TP)	N = 21 (36.8%)		
Total	N = 39 (100%)	N = 18 (100%)	N = 57 (100%)		

The p values were calculated with the Chi-square test or Fisher exact test. Boldface type illustrates statistical significance.

FU indicates follow-up, FN; false negative, FP; false positive, GRT; Gross total resection, TN; true negative, TP; true positive

### 3.5 Patient’s neurological status at last FU

At last FU, a total of 11 (20%) patients presented with CN deficits. The majority (6 patients) revealed vestibulocochlear deficits (11.1%), followed by four (7.7%) pediatric patients that showed abducens nerve palsy, three (5.8%) with optic nerve damage secondary to long-time hydrocephalus, two (3.8%) with facial hypoesthesia and 1 with facial paresis (1.9%). The most common symptom in our cohort was fine motor skills deficit seen in 16 pediatric patients (29.6%). We observed uncoordinated movement in 7 (12.7%) children, and 9 patients (16.4%) suffered from limb ataxia. Sensory deficits were seen in four (7.4%) individuals and three patients (5.5%) demonstrated motor weakness. Detailed information regarding neurological status is summarized in Table 7.

**Table 7:** Neurological Status Characteristics at last FU.

Characteristic	Frequency
Total number of patients with posterior fossa PA, n	57
Patients with a cranial nerve deficit, n (%)* 1. Optic nerve deficit 2. Abducens nerve injury 3. Trigeminal nerve injury 4. Facial nerve deficit 5. Vestibulocochlear nerve deficit	11 (20%) 3 (5.8%) 4 (7.7%) 2 (3.8%) 1 (1.9%) 6 (11.1%)
Paresis, n (%)”	3 (5.5%)
Coordination impairment, n (%)”	7 (12.7%)
Limb ataxia, n (%)”	9 (16.4%)
Intention tremor, n (%)”	5 (9.1%)
Nystagmus, n (%)^	6 (11.1%)
Gait ataxia, n (%)”	4 (7.3%)
Fine motor skills deficit, n (%)^	16 (29.6%)
Extrapyramidal symptoms, n (%)”	4 (7.3%)
Sensory deficits, n (%)^	2 (3.5%)
ADHD, n (%)^	1 (1.9%)
Intellectual disability, n (%)^	4 (7.4%)

ADHD; Attention deficit hyperactivity disorder

* 5 patients missing; ”2 patients missing; * *2 patients missing; ^3 patients missing

## 4 Discussion

PA account for ≈ 20% of all pediatric brain tumors making it one of the most common tumor entities found in children (Segal and Karajannis, 2016, Greuter et al., 2021, McKean-Cowdin et al., 2013). Being low-grade gliomas, overall survival after complete resection is excellent (Segal and Karajannis, 2016). Unfortunately, surgical management and gross-total resection of such lesions can be difficult as their most common location involves the posterior fossa. There is a paucity of studies investigating the long-term follow-up, as well as potential surgical complications after initial and especially second-look surgery after incomplete resection (Palma et al., 2004, Won et al., 2020, Collins and Pollack, 2020, Benesch et al., 2006). In addition, studies regarding partial resection and second-look surgery mainly involve small cohorts and include all brain regions (Palma et al., 2004, Benesch et al., 2006, Ait Khelifa-Gallois et al., 2015, Kristiansen et al., 2019, Kulkarni et al., 2013 Sep). Our study gives a detailed descriptive view surrounding posterior fossa PA in a large single-center pediatric population, with a special interest towards long-term postoperative outcome data, as well as outcome data after second-look surgery after partial resection.

### 4.1 Indicators of tumor progression/recurrence

Abundant evidence shows a strong correlation between tumor progression as well as recurrence, with neurological functional outcome in pediatric patients suffering from PA (Villanueva et al., 2019, Park et al., 2019, Fernandez et al., 2003, Bernhardtsen et al., 2003, Hayostek et al., 1993). Therefore, numerous studies have investigated possible predictors influencing tumor progression or recurrence. To date, the most remarkable predictor has been residual tumor mass after incomplete tumor resection (Villanueva et al., 2019, Park et al., 2019, Fernandez et al., 2003, Ogiwara et al., 2012, Bernhardtsen et al., 2003, Hayostek et al., 1993). While a small number of studies have seen opposite results and indicate that a considerable proportion of PA do not progress but rather regress after partial resection (Palma et al., 2004, Benesch et al., 2006, Dirven et al., 1997), our study found initial partial resection to be an independent predictor of tumor progression/recurrence.

### 4.2 Neurological outcome after second-look surgery

In contrast with most cohorts around PA in children that mainly investigate recurrence and overall survival (Villanueva et al., 2019, Park et al., 2019, Fernandez et al., 2003, Bernhardtsen et al., 2003, Hayostek et al., 1993, Bhatt et al., 2021), our study focused on neurological outcome after second-look surgery. This, given the above mentioned important indirect correlation between partial resection and prognosis (Villanueva et al., 2019, Fernandez et al., 2003, Ogiwara et al., 2012, Hayostek et al., 1993). We found second-look surgery to be an independent predictor of cranial nerve deficit at last FU. In 1997, Dirven et al. compared the neurological outcome between initial and second-look surgery in pediatric patients with cerebellar PA (Dirven et al., 1997). They found no significant difference between both groups. Outcome in this study was segregated into 5 groups, which was slightly similar to our mRS (that was also not significantly different between first and second-look surgery in our study). Moreover, Benesch et al. described long-term sequalae in 2 patients that went through second-look surgery after partial resection but did not provide a detailed overview of the neurological outcome (Benesch et al., 2006). Finally, as we investigated neurological outcome in a more detailed way, this considerable difference may explain our different findings.

### 4.3 Surgery of lesions located in the floor of the 4^th^ ventricle

Due to the complex anatomy of the posterior fossa, surgical treatment in this region can be technically challenging and associated with significant postoperative morbidity (Cochrane et al., 1994). The latter is more prominent when addressing lesion located in the floor of the 4th ventricle, as they contain important cranial nerve nuclei such as the vagus nerve (Ferguson et al., 2018). Our study confirmed such challenges as we found PA located in the floor of the 4th ventricle to be independently predictors of cranial nerve deficits after their resection.

### 4.4 External validity

Compared to other studies regarding posterior fossa PA in the pediatric population, our cohort seems to be representative in terms of patient characteristics. Baseline characteristics of our study compared to Ogiwara and colleagues (one of the largest series available) were similar, with 42% vs 58% females, mean age of 7.7 vs 7.3 years, most frequent location being the vermian region. Moreover, they also found extent of surgical resection to be an important predictor of tumor progression/recurrence (Ogiwara et al., 2012). This observation increases the external validity of our reported results.

### 4.5 Strengths and limitations

In the whole population, PA is a rare disease with an incidence of 8.3 per 1 million per year in children (< 15 years old) (Burkhard et al., 2003). PA in the posterior fossa have an even lower incidence, making large single-center trials difficult. Additionally, given the low rate of patients undergoing molecular analysis of the lesion, a thorough look on the outcome of patients according to their molecular status was not possible. Moreover, our data was obtained from a tertiary referral center and is not population-based, which can lead to information and selection biases. In addition, the WHO classification scheme evolved during our 20 years’ time frame, which would lead to histological bias. Prospective multicenter studies are urgently needed to validate our assumptions and to examine new predictors of patient outcome.

## 5 Conclusions

Partial resection of posterior fossa PA is a predictor for tumor progression/recurrence. Second-look surgery to remove the remnant, as well as lesions located in the floor of the 4th ventricle were predictors of cranial nerve deficit at last FU. Detailed neurological and multimodal outcome analysis is of great interest to inform families of the pros and cons of second-look surgery after partial resection.

## Conflict of interest

The authors have declared that no competing interest exists.
